# Digital five-step questionnaire to enhance standard perioperative prophylaxis in surgical patients with reported allergy to beta-lactam-antibiotics

**DOI:** 10.1017/ash.2025.10140

**Published:** 2025-09-18

**Authors:** Daniel Röder, Kathrin Eichhorn, Johanna Stoevesandt, Jan Stumpner, Patrick Meybohm, Güzin Surat

**Affiliations:** 1 Department of Anaesthesiology, Intensive Care, Emergency and Pain Medicine, University Hospital Würzburg, Würzburg, Germany; 2 Department of Dermatology, Venereology and Allergology, Allergy Center Mainfranken, University Hospital Würzburg, Würzburg, Germany; 3 Department of Anaesthesia, Intensive and Palliative Care, Klinikum Würzburg-Mitte Juliusspital, Würzburg, Germany; 4 Unit for Infection Control and Antimicrobial Stewardship, University Hospital Würzburg, Würzburg, Germany

## Abstract

**Background:**

Most self-reported beta-lactam antibiotic (BLA) allergies are inaccurate. This study evaluated a digital tool designed to reassess beta-lactam allergies and enable the use of standard perioperative antibiotic prophylaxis (PAP) without the need for prior allergy testing.

**Methods:**

In this retrospective, observational single-center cohort study, a digital five-step questionnaire was utilized during preoperative anesthesia evaluations for surgical patients reporting beta-lactam allergies. The algorithm assessed the likelihood of a beta-lactam allergy and provided recommendations for either standard PAP or the use of an alternative agent. Adherence to the algorithm’s recommendations and the incidence of allergic reactions following PAP were analyzed.

**Results:**

Between September 2020 and October 2022, 983 surgical patients reported beta-lactam allergies. Of these, 322 patients (33%) either did not receive anesthesia or did not require PAP. Among the remaining 661 patients, the algorithm recommended standard prophylaxis for 420 (64%). Of these, 262 patients received BLA, resulting in 2 allergic reactions (0.8%; negative predictive value: 99.2%), while 158 received alternative antibiotics contrary to the recommendation, leading to 3 allergic reactions (1.9%). For the 241 patients (36%) in whom the algorithm indicated a high probability of beta-lactam allergy, 197 (82%) received alternative antibiotics with 4 allergic reactions (2%). Forty-four patients (18%) received BLA contrary to the algorithm’s recommendation, with no allergic reactions observed.

**Conclusions:**

The digital five-step algorithm was a simple and effective tool during preoperative assessment, enabling safe administration of standard PAP in 64% of surgical patients with reported beta-lactam allergies.

## Introduction

Allergies to penicillin or other beta-lactam antibiotics (BLA) are reported by 8–12% of the population in Europe and North America.^1^ However, most individuals have not undergone formal testing, and over 90% of those labeled with an allergy to one or more BLAs (referred to as “BLA allergy”) are likely to tolerate re-administration of the suspected antibiotic without significant harm.^2–5^ In surgical patients, incorrect allergy labels often lead to the substitution of first-line BLAs with alternative agents for perioperative antibiotic prophylaxis (PAP). This substitution increases the risk of surgical site infections, postprocedural acute kidney injury, and the inappropriate use of broad-spectrum antibiotics.^1,6–8^ Therefore, antimicrobial stewardship programs and allergy societies worldwide emphasize the importance of systematically removing inaccurate allergy labels, particularly in non-allergist settings and among surgical patients, to promote the use of standard antibiotic therapy.^9,10^ Given the limited availability of allergists—only 10,000 specialists in Germany for approximately 1.6 million reported BLA allergies^11,12—^non-allergist-led initiatives, such as the penicillin allergy de-labeling programs established by the British Society for Allergy and Clinical Immunology ^13,14^ and the Scottish Antimicrobial Prescribing Group,^15^ are essential.

In this context, Reichel et al recently introduced a BLA de-labeling tool based solely on patient history.^7^ By answering five questions about suspected allergic reactions, the algorithm provides recommendations either for or against administering the standard BLA. Schrüfer et al retrospectively applied this tool to 800 patients previously tested for BLA allergy. The algorithm accurately identified 90% of patients with a confirmed BLA allergy and recommended de-labeling in 56% of cases where testing had already ruled out a BLA allergy.^16^

Owing to its high positive predictive value, this five-step algorithm was integrated into the electronic workflow of the preoperative anesthesiology assessment for adult surgical patients reporting a BLA allergy. The objective was to identify patients whose medical history did not indicate a genuine allergic reaction, thereby enabling the administration of recommended first-line BLAs without prior allergy testing. Recommendations for or against standard PAP administration were documented in the patients’ electronic health records, with the final decision left to the attending anesthesiologist.

This study presents data on the feasibility and safety of digitally implementing the algorithm below (Figure 2) into the preoperative anesthesiology workflow. It is the first study to apply a history-based digital tool during perioperative assessment to reevaluate reported BLA allergies and enhance the use of standard PAP.

## Methods

### Study design and patients

This retrospective, observational single-center cohort study was conducted at the University Hospital Würzburg (Würzburg, Germany), a 1,500-bed tertiary hospital. The local institutional review board approved the retrospective review and publication of anonymized clinical data (Ref.no. 20221124 01).

**Figure 1.**
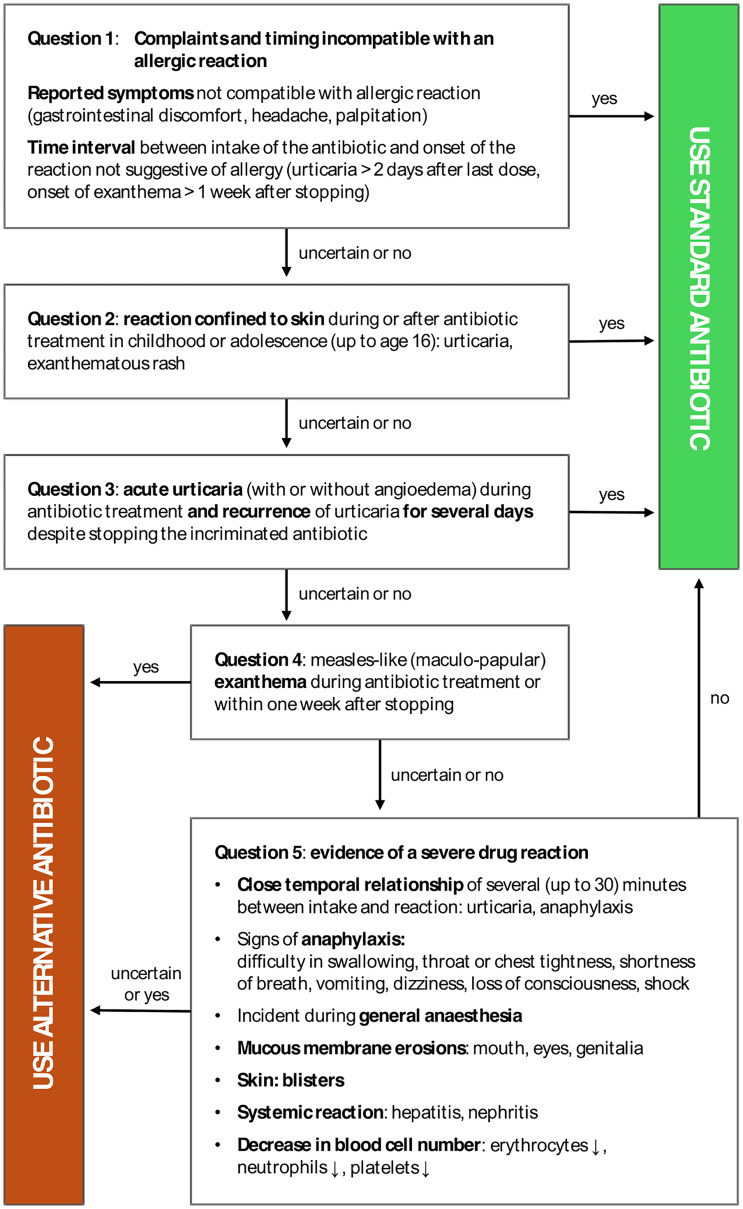
Beta-lactam antibiotic allergy revaluating tool, modified after Reichel (7).

In July 2020, a BLA allergy revaluating tool (Figure 1)—a five-step-questionnaire generating algorithm-derived recommendations (“STANDARD” or “ALTERNATIVE”)—became a routine component of preoperative anesthesiology assessments. The tool was implemented as part of the local antimicrobial stewardship program to promote the use of appropriate first-line antibiotics for perioperative prophylaxis, consistent with current guidelines.^17,18^ The mandatory questionnaire was integrated into the electronic anaesthesiologic assessment form, showing up automatically once a Penicillin allergy was recorded, with patient responses and algorithm recommendation documented in their electronic health records (IS-H/i.s.h.med under SAP, Cerner Corporation, North Kansas City, MO, USA, and SAP SE, Walldorf, Germany).

### Data collection

The study included adult patients (≥18 yrs) scheduled for elective surgery who reported a BLA allergy during their preoperative anesthesia evaluation between September 20, 2020, and October 31, 2022. No exclusion criteria were applied. Data were retrospectively extracted from the local patient data management systems (COPRA6 RM1.0, COPRA System GmbH, Berlin, Germany, and IS-H) and included the algorithm’s recommendations, the antibiotic administered before skin incision and vital signs before and after administration. Anesthesia records and discharge letters were reviewed for signs of immediate or delayed allergic reactions within 48 h postsurgery.

### Questionnaire administration

The digital questionnaire was completed by the attending anesthesiologist during the preoperative consultation. The algorithm was accessible to all clinicians, but only the anesthesiologists had the authority to actively use it. In cases where patient responses were unclear (eg, distinguishing urticaria from other rashes), the anesthesiologist sought clarification directly from the patient and reviewed available medical records. Anesthesiologists tasked with protocol implementation were trained by allergy specialists and subsequently trained their colleagues on the proper use of the questionnaire for reassessment of reported BLA allergies.

### Definition of an allergic reaction during anesthesia

Heart rate, systolic blood pressure, norepinephrine dosage, peripheral oxygen saturation, and in mechanically ventilated patients, end-tidal partial pressure of carbon dioxide and peak inspiratory pressure were monitored immediately before and within 15 minutes after the administration of PAP. The possibility of allergic reactions to other perioperatively administered drugs or substances (eg, anesthetics, muscle relaxants) was evaluated by reviewing detailed anesthesia records. Additional allergy-specific tests, such as serum tryptase levels or skin testing, were not performed to confirm or rule out alternative causes.

#### Immediate-type allergic (Anaphylactic) reaction

- Cutaneous symptoms (eg, flushing, urticaria, angioedema).
- Dyspnea (eg, cyanosis, wheezing, attenuated or absent breath sounds) or bronchoconstriction (eg, need for bronchospasmolytics, peripheral oxygen saturation <92%, peak inspiratory pressure >20 cmH_2_O or increased by >10 cmH_2_O from baseline, end-tidal CO_2_ >45 mmHg).
- Hypotension (systolic blood pressure <90 mmHg or a decrease >30% from baseline; norepinephrine dosage increase >30% from baseline).
- Tachycardia (heart rate >100 beats per minute or an increase >30% from baseline) up to full-blown distributive shock.

#### Delayed-type reaction

Maculopapular or macular rash, pustular or bullous reactions, with or without systemic symptoms, occurring 48–72 hours after the suspected antibiotic was administered.

### Statistical analysis

A data analysis and statistical plan was written after data were accessed. Categorical variables are presented as absolute numbers and percentages, while continuous variables are expressed as medians with interquartile ranges (IQR, 25–75%). Categorical data were analyzed using the *X*^2^ test. A *p*-value of ≤0.05 was considered statistically significant. Data were analyzed using Microsoft Excel^®^ 2016 (Microsoft Corporation, Redmond, WA, USA) and IBM SPSS Statistics, Version 28.0 (IBM Corporation, Armonk, NY, USA).

## Results

**Figure 2.**
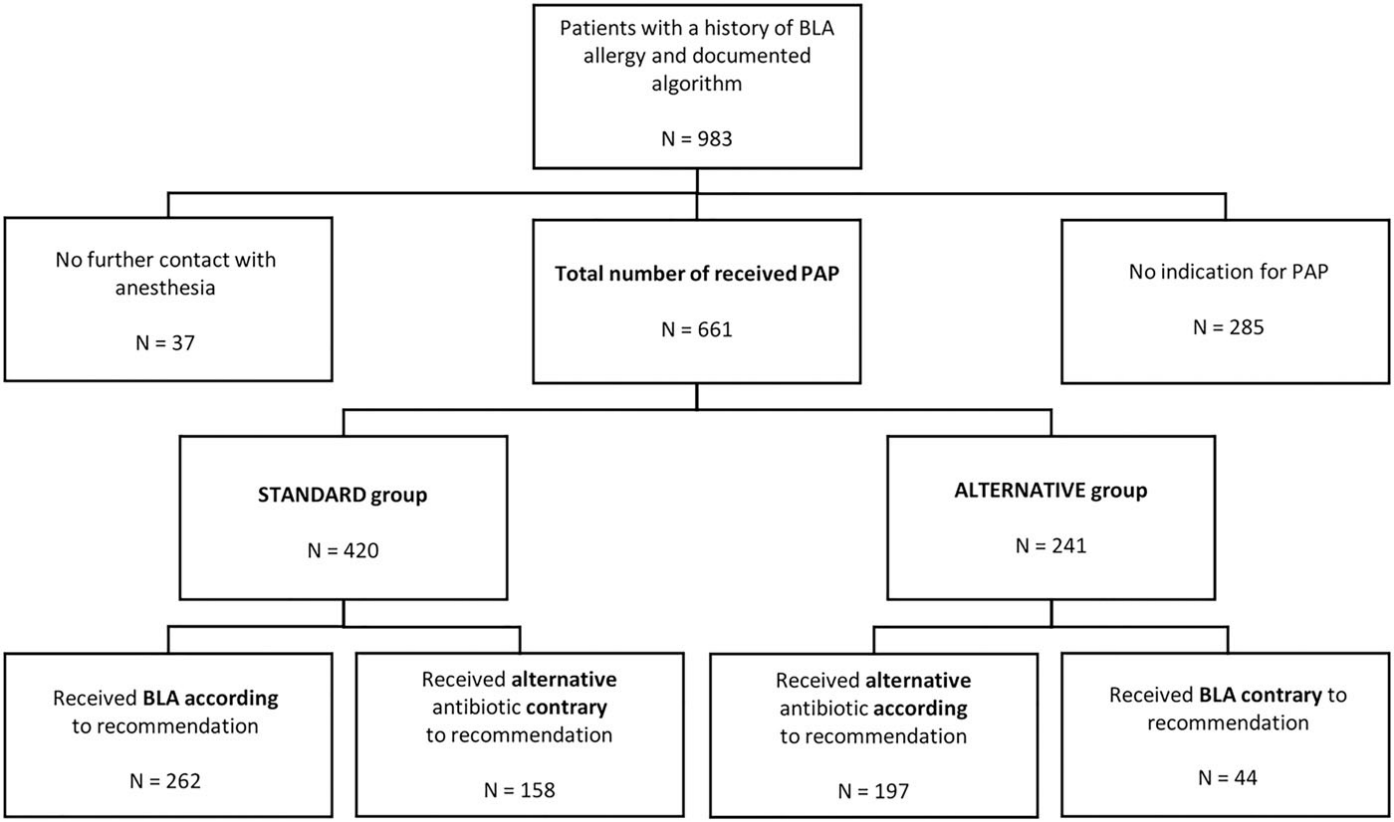
Flowchart of patient enrolment.

We analyzed data from 983 patients who reported a history of BLA allergy and completed the questionnaire. Of these, 661 patients received PAP, while 322 were excluded due to either a lack of indication for PAP during surgery (*n* = 285) or no subsequent contact with the anesthesia team (*n* = 37; Figure 2). The characteristics of the 661 patients included in the study are summarized in Table 1. Table 2 provides details on the antibiotics administered and the occurrence of allergic reactions. The algorithm recommended standard PAP in 420 patients (64%; STANDARD group). Of these, 262 patients received BLA, including ampicillin/sulbactam (*n* = 25), cefazolin (*n* = 227), cefuroxime (*n* = 1), penicillin (*n* = 4), and piperacillin/tazobactam (*n* = 5). However, 158 patients received an alternative antibiotic contrary to the algorithm’s recommendation, including ciprofloxacin (*n* = 2), clindamycin (*n* = 147), gentamicin (*n* = 2), and vancomycin (*n* = 7). In the STANDARD group, two patients experienced mild allergic reactions following cefazolin administration. These included flushing during surgery without further systemic symptoms and a benign skin rash on the first postoperative day, which appeared related to metamizole administration and resolved after its discontinuation. Additionally, three patients developed circulatory or pulmonary symptoms after clindamycin administration, including increased heart rate and a drop in systolic blood pressure (*n* = 2) and bronchospasm four hours after endotracheal extubation (*n* = 1). Overall, 260 of 262 patients in the STANDARD group exhibited no signs of allergic reaction, yielding a negative predictive value of 99.2%. The positive predictive value of the algorithm could not be determined, as patients with suspected BLA allergies did not undergo confirmatory testing.

**Table 1.** Patient characteristics (age, sex, surgical discipline)

	Total (*n* = 661)	STANDARD (*n* = 420)	ALTERNATIVE (*n* = 241)
**Age** (years)	55 (42,66 [19–93])	54 (41,66 [19–92])	57 (43,68 [20–93])
**Sex**			
Female (%)	445 (67%)	269 (64%)	176 (73%)
Male (%)	216 (33%)	151 (36%)	65 (27%)
**Surgical discipline**			
Cardiothoracic (%)	72 (11%)	45 (11%)	27 (11%)
Ear, nose, throat (%)	59 (9%)	38 (9%)	21 (9%)
General/Visceral (%)	176 (27%)	110 (26%)	66 (27%)
Neurosurgery (%)	55 (8%)	36 (9%)	19 (8%)
Obstetrics/Gynecology (%)	72 (11%)	32 (8%)	40 (17%)
Ophthalmology (%)	1 (0%)	1 (0%)	0 (0%)
Oral, maxillofacial (%)	33 (5%)	19 (5%)	14 (6%)
Trauma (%)	121 (18%)	87 (21%)	34 (14%)
Urology (%)	72 (11%)	52 (21%)	20 (8%)

Data are numbers (percentage) and median (quartiles [range]).

**Table 2.** Administered antibiotics and allergic reactions

	Total	Allergic Reactions	STANDARD	Allergic Reactions	ALTERNATIVE	Allergic Reactions
**Total**	**661**	**9 (1.4%)**	**420**	**5 (1.2%)**	**241**	**4 (1.7%)**
**Received BLA (%)**	**306 (46.3%)**	**2 (0.7%)**	**262 (62.4%)**	**2 (0.8%)**	**44 (18.3%)**	**0**
Ampicillin/Sulbactam	27 (4.1%)		25 (6.0%)		2 (0.8%)	
Cefazolin	265 (40.1%)	2 (0.3%)	227 (54.0%)	2 (0.5%)	38 (15.8%)	
Cefuroxime	1 (0.2%)		1 (0.2%)		0	
Flucloxacillin	1 (0.2%)		0		1 (0.4%)	
Meropenem	1 (0.2%)		0		1 (0.4%)	
Penicillin	4 (0.6%)		4 (1.0%)			
Piperacillin/Tazobactam	7 (1.1%)		5 (1.2%)		2 (0.8%)	
**Received alternative (%)**	**355 (53.7%)**	**7 (2.0%)**	**158 (37.6%)**	**3 (1.9%)**	**197 (81.7%)**	**4 (2%)**
Ciprofloxacin	5 (0.8%)		2 (0.5%)		3 (1.2%)	
Clindamycin	322 (48.7%)	6 (0.9%)	147 (35.0%)	3 (0.7%)	175 (72.6%)	3 (1.2%)
Cotrimoxazole	1 (0.2%)		0		1 (0.4%)	
Gentamicin	7 (1.1%)	1 (0.2%)	2 (0.5%)		5 (2.1%)	1 (0.4%)
Metronidazole	2 (0.3%)		0		2 (0.8%)	
Vancomycin	18 (2.7%)		7 (1.7%)		11 (4.6%)	

Data are numbers (percentage), no significant differences in any of these groups.

The algorithm recommended the use of an alternative antibiotic in 241 patients (36%; ALTERNATIVE group). This included 124 patients reporting a measles-like exanthema (positive response to question 4) and 117 patients with potentially severe delayed-type or immediate-type reactions/anaphylaxis (positive response to question 5). In the ALTERNATIVE group, 197 patients received alternative antibiotics, including ciprofloxacin (*n* = 3), clindamycin (*n* = 175), cotrimoxazole (*n* = 1), gentamicin (*n* = 5), metronidazole (*n* = 2), and vancomycin (*n* = 11). Conversely, 44 patients received BLA contrary to the recommendation, including ampicillin/sulbactam (*n* = 2), cefazolin (*n* = 38), flucloxacillin (*n* = 1), meropenem (*n* = 1), and piperacillin/tazobactam (*n* = 2).

No allergic reactions were observed in any of the 44 patients who received standard BLA prophylaxis in the ALTERNATIVE group. However, four patients (2%) experienced allergic reactions after receiving alternative antibiotics. One patient developed hypotension immediately after clindamycin administration, which could not be attributed to other anesthetic or surgical factors. Three patients exhibited cutaneous symptoms, including exanthema after gentamicin (*n* = 1), pruritus after clindamycin (*n* = 1), and delayed exanthema after clindamycin (*n* = 1).

Overall, the algorithm’s recommendations were followed in 69% of patients (459/661), with significantly higher adherence in the ALTERNATIVE group compared to the STANDARD group (82% vs 62%, *P* < 0.001). Symptoms indicative of an allergic reaction were observed in nine patients (1.4%), but no cases of anaphylactic shock occurred in either group.

## Discussion

This study evaluated the feasibility and safety of a digital five-step questionnaire designed to support the use of first-line PAP in surgical patients reporting an allergy to one or more BLA. To our knowledge, this is the first digitally based program to reevaluate BLA allergies outside the dermatological domain, using patient history during preoperative anesthesia evaluations to enable the use of standard PAP without prior allergy testing.

### De-labeling versus revaluating

The term “allergy de-labeling” refers to the removal of allergy labels from patients’ health records, typically following allergy testing. In this retrospective study, no allergy labels were formally removed, and patients were not advised to refrain from reporting a BLA allergy in the future. Instead, their reported allergy was revaluated based on the questionnaire results. In the STANDARD group, first-line BLA were administered despite the reported allergy. Therefore, we believe our findings merit discussion within the context of de-labeling studies.

### Feasibility and implementation

The digital revaluation tool identified 64% of patients as low-risk for BLA allergy, recommending standard beta-lactam prophylaxis. This proportion significantly exceeds the 14% success rate reported in a recent meta-analysis, where non-allergists de-labeled patients based on history alone.^19^ However, adherence to the algorithm’s recommendations did not exceed 69%, highlighting the challenges of translating recommendations into practice. Reluctance among anesthesiologists may stem from safety concerns or insufficient knowledge about antibiotic allergy principles. Targeted education on antibiotic stewardship and allergy management could improve adherence and confidence in the tool’s recommendations.

### Safety and cross-reactivity

Our study demonstrated a negative predictive value of 99.2% (260/262 STANDARD patients), consistent with reported rates of 84.9% to 98.4% in previous studies.^20–22^ Two patients in the STANDARD group exhibited mild reactions: one experienced a drop in systolic blood pressure (103 mmHg to 89 mmHg) within 10 minutes of cefazolin infusion, and another developed an itchy truncal rash on the first postoperative day, likely associated with metamizole. These reactions cannot be conclusively attributed to the antibiotics due to the complexity of perioperative factors, including anesthesia agents and surgical conditions. Importantly, no severe allergic reactions or anaphylactic shocks were observed.

Our findings align with previous studies supporting the safe use of cefazolin in patients with reported BLA allergies.^23^ Cefazolin’s unique side chain on the beta-lactam ring minimizes cross-reactivity with penicillins, with a true allergy rate of under 1% between penicillin and cefazolin.^24^ In this study, 38 patients in the ALTERNATIVE group received cefazolin despite reported BLA allergies. No allergic reactions were observed, which supports the safe use of cefazolin as a first-line PAP for a wide range of surgical procedures. However, tolerance to cefazolin does not guarantee tolerance to other beta-lactams, underscoring the need for caution in overgeneralising de-labeling outcomes.

### Specificity

Given the low prevalence of confirmed BLA allergies (0.5–2%),^2,16,25^ the algorithm’s specificity appears limited, as it recommended alternative antibiotics for over one-third of patients. However, this approach aligns with the high safety standards required in this context, prioritising the prevention of harm to genuinely allergic patients. While this may result in more patients receiving alternative antibiotics than strictly necessary, it ensures that the majority can still safely receive standard antibiotics. Notably, achieving standard PAP in over 60% of patients without prior testing represents a significant improvement compared to the 14% success rate reported in a recent meta-analysis.^19^

### Limitations and future directions

This study has several limitations. First, its retrospective design and single-center setting limit the generalizability of the findings. Second, patients the algorithm denied de-labeling occasionally tolerated beta-lactams, suggesting that the algorithm’s criteria could be refined. Third, immediate allergic reactions may have been overestimated due to confounding perioperative factors, while delayed reactions may have been underreported due to the lack of systematic follow-up. This trial was meant as a pilot phase to confirming the safe administration of cefazolin too, and to laying the foundation for an in-house de-labeling concept. We did not proceed with de-labeling measures as it involves more key-players (integration of A and E) and a wider strategic approach. Finally, the algorithm has not been formally validated outside the perioperative setting, limiting its applicability.

It is important to note that de-labeling based solely on patient history carries a residual risk of recurrent allergic reactions. As such, de-labeled patients should only receive beta-lactams under medical supervision.^6^ The perioperative setting provides ideal conditions for medically supervised re-exposure, with anesthesiologists present, intravenous access available, and continuous vital sign monitoring.

Our experiences and results will be integrated in our future in-house roll-out, hoping to increase the user-friendliness of the questionnaire by optimizing the circumstances and the tool itself.

Future studies should incorporate systematic follow-up protocols after removal of allergy labels from the patient’s health records. Additionally, the long-term impact of this approach on healthcare utilization and antibiotic stewardship should be explored.

## Conclusion

The presented digital five-step revaluation tool, based solely on patient history, is both feasible and practical for use by non-allergists. It facilitates the administration of first-line PAP in nearly two-thirds of adult surgical patients with a reported allergy to one or more BLA, without causing severe allergic reactions. Moving forward, anesthesiologists should take a more active role in supporting international recommendations to systematically remove incorrect allergy labels, thereby promoting the use of standard PAP.
